# Conditioned stimulus presentations alter anxiety level in fear-conditioned mice

**DOI:** 10.1186/s13041-019-0445-4

**Published:** 2019-03-29

**Authors:** Yujie Zhang, Kunfu Ouyang, Tatiana V Lipina, Hong Wang, Qiang Zhou

**Affiliations:** 1 State key laboratory of chemical oncogenomics, School of Chemical Biology and Biotechnology, Peking University Shenzhen Graduate School, Shenzhen, China; 2 Federal State Budgetary Scientific Institution, Scientific Research Institute of Physiology and Basic Medicine, Novosibirsk, Russia; 3 University of Toronto, Department of Pharmacology & Toxicology, Toronto, Ontario Canada

**Keywords:** Discriminative fear conditioning, Diazepam, Amygdala, PKMzeta, Heart rate, Calcium imaging, Ventral hippocampal CA1

## Abstract

It is generally believed that fear is rapidly triggered by a distinct cue while anxiety onset is less precise and not associated with a distinct cue. Although it has been claimed that both processes can be measured with certain independence of each other, it is unclear how exactly they differ. In this study, we measured anxiety in mice that received discriminative fear conditioning using behavioral, heart rate and calcium (Ca^2+^) responses in the ventral hippocampal CA1 (vCA1) neurons. We found that the occurrence of fear significantly interfered with anxiety measurements under various conditions. Diazepam reduced basal anxiety level but had no effect during the presentation of conditioned stimulus (CS). Injection of an inhibitory peptide of PKMzeta (ZIP) into the basolateral amygdala almost entirely abolished CS-triggered fear expression and reduced anxiety to basal level. Heart rate measures suggested a small reduction in anxiety during CS-. Calcium responses in the lateral hypothalamus-projecting vCA1 neurons showed a steady decay during CS suggesting a reduced anxiety. Thus, under our experimental conditions, CS presentations likely reduce anxiety level in the fear-conditioned mice.

**Electronic supplementary material:**

The online version of this article (10.1186/s13041-019-0445-4) contains supplementary material, which is available to authorized users.

## Introduction

In both literatures of clinical psychiatry and basic neuroscience, the distinction between anxiety and fear is ambiguous, and often one is used to define the other [1]. One prevailing view is that fear is specific to responses directed to a present threat (i.e., when facing a threat) associated with specific cues (such as a sound that predicts the incoming of a foot shock), while anxiety is in preparation for threats of future-oriented [2]. In this regard, fear is directed at something specific and concrete, while anxiety to something diffuse and abstract in nature. Evidences suggest that a standard extinction procedure with repeated expose to non-reinforced conditioned stimulus (CS) may reduce fear responses to the CS, but anxiety associated with or caused by the fearful experience remains [3]. Anxiety disorder is one of the most prevalent psychiatric disorders, the lifetime prevalence of human population is up to 30%, and is also highly comorbid with other mental disorders [4].

Since anxiety is highly associated with various psychiatric diseases (such as post-traumatic stress disorder and phobia), a better understanding of this question using animals is of great importance. From a technical point of view, Pavlovian fear conditioning is well-established in animals, and fear expression (usually measured with freezing) can be readily quantified. Measurement of anxiety, on the other hand, is less well-defined, especially in animals. In humans, anxiety can be measured based on the accounts from subjects (as whether they feel anxious) and by various autonomic responses, such as changes in heart rate and blood pressure [5–8]. In rodents, most widely used measurements of anxiety include open field test (OFT), elevated plus maze (EPM) and light/dark chamber shuttle box [9, 10]. These measurements make intuitive sense since rodents tend to avoid potentially threating or dangerous stimuli (such as bright light) and open areas because these typically signal potential threats or areas which make them more vulnerable to attack. Hence, anxiety measurement using these approaches is generally regarded as innate anxiety. Anxiety levels measured by OFT, EPM and shuttle box are sensitive to amelioration by benzodiazepine type of anxiolytics [10–13]. Ventral hippocampus (vHPC) is highly implicated in anxiety [14–16]. Recently, it has been reported that the majority of vCA1 neurons showed significantly increased Ca^2+^ activity when mice entering the open arm on a EPM, and silencing vCA1 neurons projecting to lateral hypothalamus area (LHA) significantly increased open arm time [17]. Thus, Ca^2+^ responses during CS presentation in vCA1-LHA neurons may be a direct measure of anxiety level.

In this study, we asked a simple question: can CS induce measurable changes in the anxiety level in mice received auditory fear conditioning? We used various measurements of anxiety in mice, together with measurement of fear responses. We found some evidence that anxiety level is, actually, reduced during CS presentation in fear-conditioned mice.

## Methods

### Animals

C57BL/6J wild-type mice were purchased from Guangdong Medical Laboratory Animal Center. Only male mice of 9–12 week were used. Mice were maintained in a pathogen-free temperature-controlled (22 ± 1 °C) mouse facility and had access to food and water ad libitum*.* Mice were housed on a reversed 12 h light-dark cycle (light on at 8:00 AM.) with 5–6 mice per cage, at Peking University Shenzhen Graduate School. All behavior experiment performed between 9:00 AM and 6:00 PM.

### Fear conditioning

Differential auditory fear conditioning was used to induce distinct responses to CS+ and CS-. For conditioning, after 3-min habituation in the training cage (Coulbourn Instruments; context A), mice were subjected to 6 trials of tone. Three tones (3 kHz, 70 dB, amplitude is 100, rise time is 5, and Rep rate is 2 Hz, duty cycle is 50%, as CS+) were co-terminated with a foot shock (0.8 mA, 2 s) while three tones (white noise, as CS-) were not paired with foot shock. Each tone lasted for 30 s, and they were presented in pseudo-random order with a 90 s intertrial interval (ITI). The training chamber was wiped with 1% acetic acid before the next experiment. Fear memory retention was tested in a novel context (context B) with a different shape (35*20*20 cm) compared to the training context (context A). During recall test, 3 min was given prior to two CS- and two CS+. Testing chamber was wiped with 75% alcohol before next experiment. FreezeFrame software was used to control the delivery of tones and foot shocks. Freezing time was calculated using the same software. Each cage was placed inside a sound-attenuated chamber.

### Shuttle box test

Shuttle box consisted of 2 compartments with different illumination intensity: a light chamber (295 lx) and a dark chamber (0 lx). They were of the same size and shape (21*21*25 cm) and separated by a Plexiglas wall (21*25 cm). A hole of 3 × 5 cm at the bottom of separating wall connected the two chambers. Mice were allowed to move freely between these two chambers. Shuttle box was placed inside the conditioning chamber. At beginning of the test, mice were placed inside the dark chamber. After 5 min of free exploration, two 30s CS- were presented followed by two 30s CS+ and the ITI were 90 s. Locomotion was recorded using a camera placed above the shuttle box, and time spent by each mouse in the light box was measured manually. Shuttle box was cleaned with 30% isopropanol after each test.

### Elevated plus maze (EPM) test

The EPM apparatus consisted of two open arms (30*5 cm), two closed arms of the same size with 15 cm high walls and a center platform (5*5 cm). The apparatus was elevated to a height of 35 cm above the test room floor. Mice were placed in the test room to habituate for 1–2 h. Mice were placed in the central area facing one of the open arms. Time in open arm and number of entries to open arm were recorded during 300 s automatically using ANY-maze software. The apparatuses were cleaned with 75% alcohol after each test. During the tone-EPM test, after 300 s of baseline testing, recorded tone was played through a recorder, with two 30s CS- were presented followed by two 30s CS+ and the ITI were 90 s. For experiments testing the effects of CS on parameters on the EPM, average of these parameters in every minute was used.

### Open field test (OFT)

Open field test was performed in a 50*50 *50 cm chamber. Mice were placed in the center zone of OFT and allowed to move freely for 15 min, with their movement recorded using ANY-maze software. The chamber was cleaned with 75% alcohol between each test. Total distance traveled and times spent in the center area were analyzed using ANY-maze software.

### In vivo Ca^2+^ measurement

For in vivo Ca^2+^ imaging, mice underwent a single surgery. Mice were deeply anesthetized with isoflurane and injected with virus prior to implanting the ceramic ferrule containing the optical fiber (230 μm O.D., 0.37 numerical aperture (NA); Shanghai Fiblaser) over the injection site. Three hundred nl of retrograde rAAV-hSyn-Cre-WPRE-pA virus (Brain VTA Technology Co. Ltd., China) at an injection speed of 80 nl/min was injected bilaterally in the lateral hypothalamus area (LHA; − 1.7 AP, ±0.9 ML, − 5.8 DV; from Bregma). Five hundred nl of rAAV-Ef1α-DIO-GCaMP6s-WPRE-pA virus (Brain VTA Technology Co. Ltd., China) was injected in the vCA1 (− 3.16 AP, 3.55 ML, − 4.8 DV; from Bregma) unilaterally using a microsyringe pump. After virus injection, 2 skull screws were inserted around the implantation site, and ceramic ferrule was slowly lowered into vCA1 (− 3.16 AP, 3.55 ML, − 4.8 DV; from Bregma) and fixed to the skull with dental acrylic. Imaging experiments were conducted 4 weeks after this procedure. To record fluorescence signals, laser beam from a 473 nm LED was reflected by a dichroic mirror (MD498; Thorlabs), focused by a 20 x objective lens (NA = 0.4; Olympus). An optical fiber (230 μm O.D., NA = 0.37, 2 m long) was used. To minimize bleaching, laser power at the tip of optical fiber was set to 20~25 μW. GCaMP fluorescence signal was band-pass filtered (MF 525–39, Thorlabs) and collected using CMOS cameras (DCC3240M, Thorlabs). The CMOS convert the fluorescence signal to digital signal. Signals were sent to computer and digitalized at 50 Hz and recorded by a multi-channel fiber photometry recording system (Thinker Tech). Fiber-photometry recording data were analyzed using MATLAB.

### Heart rate measurements

One week before surgery, mice were housed individually. Each mouse was implanted with a radio-telemetry transmitter (model TA11ETA-F10, Datasciences, St. Paul, MN, USA). On the day of surgery, mice were deeply anesthetized with isoflurane. The transmitter body with two recording leads were placed and fixed in place in the right side of abdomen. One lead was guided subcutaneously to the neck and fixed in position with muscle tissue, while the other lead was placed to the opposite of the abdomen. One week recovery was allowed after surgery. Before fear conditioning, mice were habituated to the tone in home cage for 2 days to avoid the impact of tone on HR. Mice were trained using the same protocol. Memory retention and Heart rate (HR) were tested 24 h after conditioning in the home cage. After 10 min of habituation, two CS- and two CS+ were given. Locomotion was recorded by a camera and freezing was calculated manually. Heart rate and R-R intervals (beat-to-beat interval) were continuously recorded during testing at a sampling rate of 500 Hz using Dataquest ATR 4.33 software (Datasciences, St. Paul, MN, USA). For statistical analysis, HR and RR intervals were averaged every 5 s.

### Drug infusion

On the day of surgery, mice were deeply anesthetized with isoflurane. The stereotaxic coordinates for basolateral amygdala (BLA) were AP − 1.4 mm and ML ±3.47 mm and DV 5.08 mm. The PKMzeta inhibitor ZIP was dissolved in sterile saline and infused at a concentration of 10 mM. ZIP or saline were infused into BLA (400 nl per hemisphere) at a rate of 80 nl/min. The injection needle was left in place for an additional 5 min. Diazepam was dissolved in sterile saline and injected (i.p) at a dose of 1.5 mg/kg 30 min before behavioral testing (EPM, OFT or shuttle box).

### Data analysis

Data were analyzed using GraphPad Prism software. Statistical analysis was performed using unpaired t-test, paired t-test, One-way or Two-way Repeated Measures ANOVA (One-way or Two-way RM ANOVA) followed by Bonferroni post-test which were specifically stated in the Results. All results were shown as Mean ± S.E.M. *P* < 0.05 was considered statistically significant.

## Results

### Anxiety associated with conditioned stimulus after discriminative fear conditioning

**Fig. 1:**
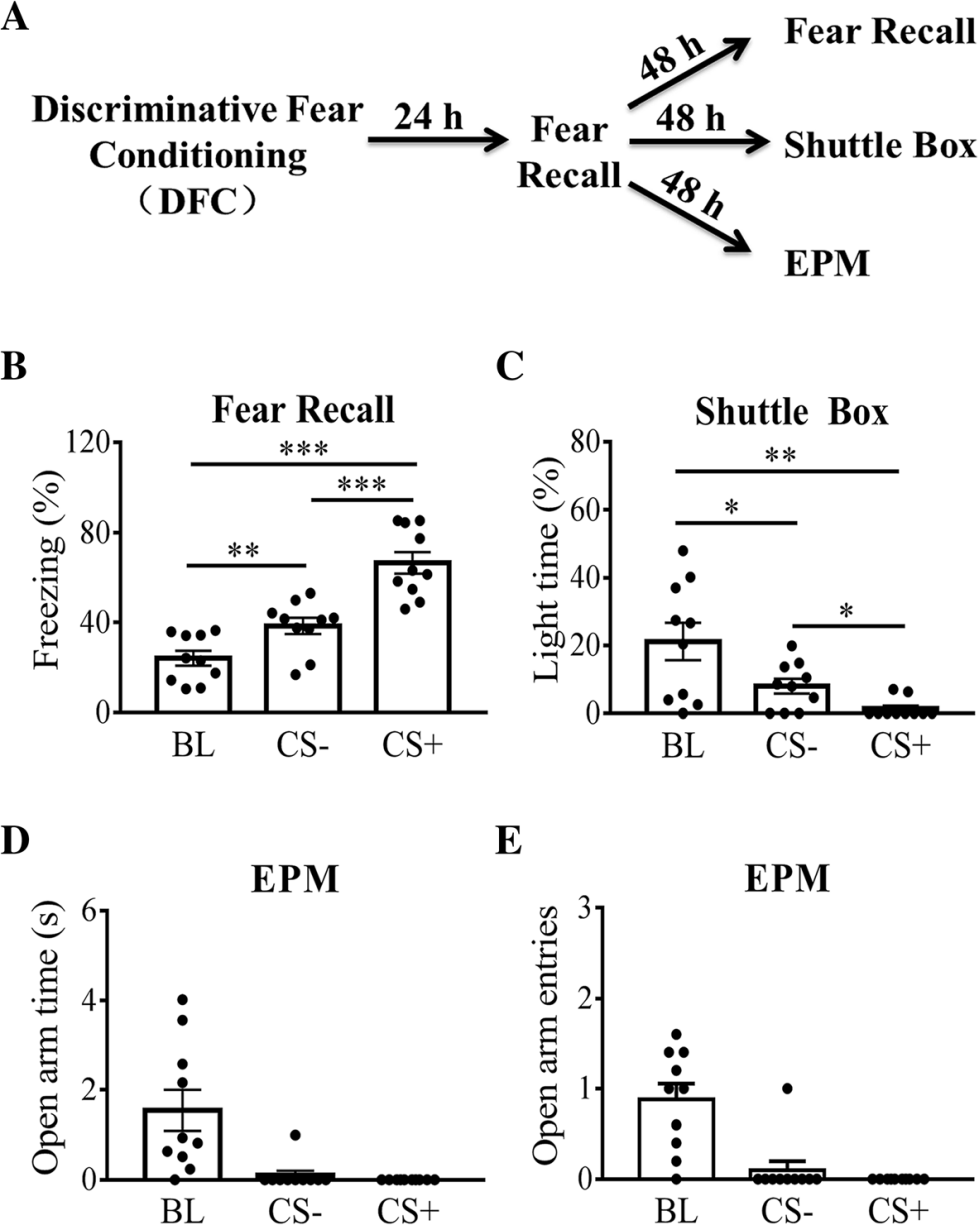
Anxiety measurements during the recall of fear memory. **a** Experimental procedures. Twenty-four hours after CS+/CS- discriminative fear conditioning, mice were tested on memory retention. They were then divided into three groups with comparable freezing levels. In 48 h, one group was tested for fear memory (*n* = 10), one tested in shuttle box (*n* = 10) and the third group on EPM (*n* = 10). **b** Freezing levels during fear recall in context B (48 h after fear retention test). **c** Light time (%) in the shuttle box during CS presentation. **d** Open arm time on the EPM during CS presentation. **e** Number of open arm entries on the EPM during CS presentation. *, *p* < 0.05; **, *p* < 0.01; ***, *p* < 0.001. Data were analyzed by One-way RM ANOVA followed by Bonferroni post-test

The aim of these experiments was to test whether presentation of CS (sound) can alter anxiety level in mice that have received discriminative fear conditioning (Fig. 1a). Two most commonly used tests of anxiety, light/dark shuttle box and EPM, were used. Fear responses were measured with freezing levels. Discriminative fear conditioning was used so that CS+ and CS- were associated with different freezing levels, and hence we can test the relationship between anxiety and fear levels.

As shown in Fig. 1b, CS affected freezing level significantly (F _(2, 18)_ = 57.87, *p* < 0.0001; One-way RM ANOVA followed by Bonferroni post-test). We found that CS increased freezing levels in the non-conditioning context (context B) compared to the basal level (BL, a 3 min habituation period prior to CS presentation, CS- vs. BL, *p* < 0.01; CS+ vs. BL, *p* < 0.001; Bonferroni post-test). The increase in freezing level was higher during CS+ than during CS-, confirming the efficacy of differential fear conditioning (CS- vs. CS+, *p* < 0.001). In the light/dark shuttle box, One-way RM ANOVA followed by Bonferroni post-test also revealed that CS affected light time significantly (Fig. 1c; F _(2, 18)_ = 12.69, *p* < 0.01), and there was a significant reduction in light time for both CS- and CS+ (Fig. 1c, CS- vs. BL, *p* < 0.05; CS+ vs. BL, *p* < 0.01; Bonferroni post-test). There was a significant difference in light time between CS+ and CS- (CS- vs. CS+, *p* < 0.05).

As a second measurement of anxiety, we examined changes on the EPM in mice that had not been tested in the shuttle box to avoid potential interactions/interference between the two tests. CS induced a significant reduction in the open arm time (Fig. 1d, f _(2, 18)_ = 11.98, *p* < 0.01; One-way RM ANOVA) and open arm entries (Fig. 1e, f _(2, 18)_ = 15.23, *p* < 0.01). Since most values of open arm time or open arm entries during CS were 0, we did not perform any additional analysis. In addition, we also found that CS induced significant changes in other parameters in EPM when compared to BL (Additional file 1; Table S1; One-way RM ANOVA followed by Bonferroni post-test). In summary, these results indicate that CS triggers a rapid change in freezing level, light time and open arm times or entries. However, it is confused that CS- as a safety signal, did not reduce anxiety level (light time or open arm time) compared to baseline.

### Effect of diazepam (DZP) on anxiety measurements during CS presentation

The results in Fig. 1 suggest that there could be changes in anxiety level (light time and open arm time/entries) during CS presentation. However, we cannot exclude the possibility that these changes were most likely influenced or even caused by the occurrence of fear/freezing. For example, CS-induced freezing could cause mice to stay in the dark chamber of the shuttle box during CS and hence significantly reduce their time in the light chamber. In other words, these changes in anxiety could mainly reflect fear rather than anxiety. To address this possibility, we asked whether a drug known to reduce anxiety level can modulate these parameters. Plenty of evidences suggest that diazepam (DZP), an allosteric GABAa receptor enhancer, can effectively do so [10–13].

Consistent with prior findings, i.p injection of diazepam prior to testing increased open arm time (Additional file 2; Figure S1A, 1B; two-tailed unpaired t test, T_25_ = 3.03, *p* < 0.01) and open arm entries (Additional file 2; Figure S1C; two-tailed unpaired t test, T_25_ = 3.49, *p* < 0.01) in fear-conditioned mice, in the absence of CS. In addition, we also found diazepam affect other parameters significantly in EPM (Additional file 1: Table S2; two-tailed unpaired t test). In contrast, the same injection did not cause any significant changes in light time (Additional file 2: Figure S1D, T_24_ = 0.20, *p* > 0.05), or center time in the open field test (Additional file 2; Fig. S1E, T_24_ = 1.45, *p* > 0.05). Total distance travelled in OFT was also unaffected by diazepam (Additional file 2; Fig. S1F; T_24_ = 1.47, *p* > 0.05), suggesting no effect on locomotion.

**Fig. 2:**
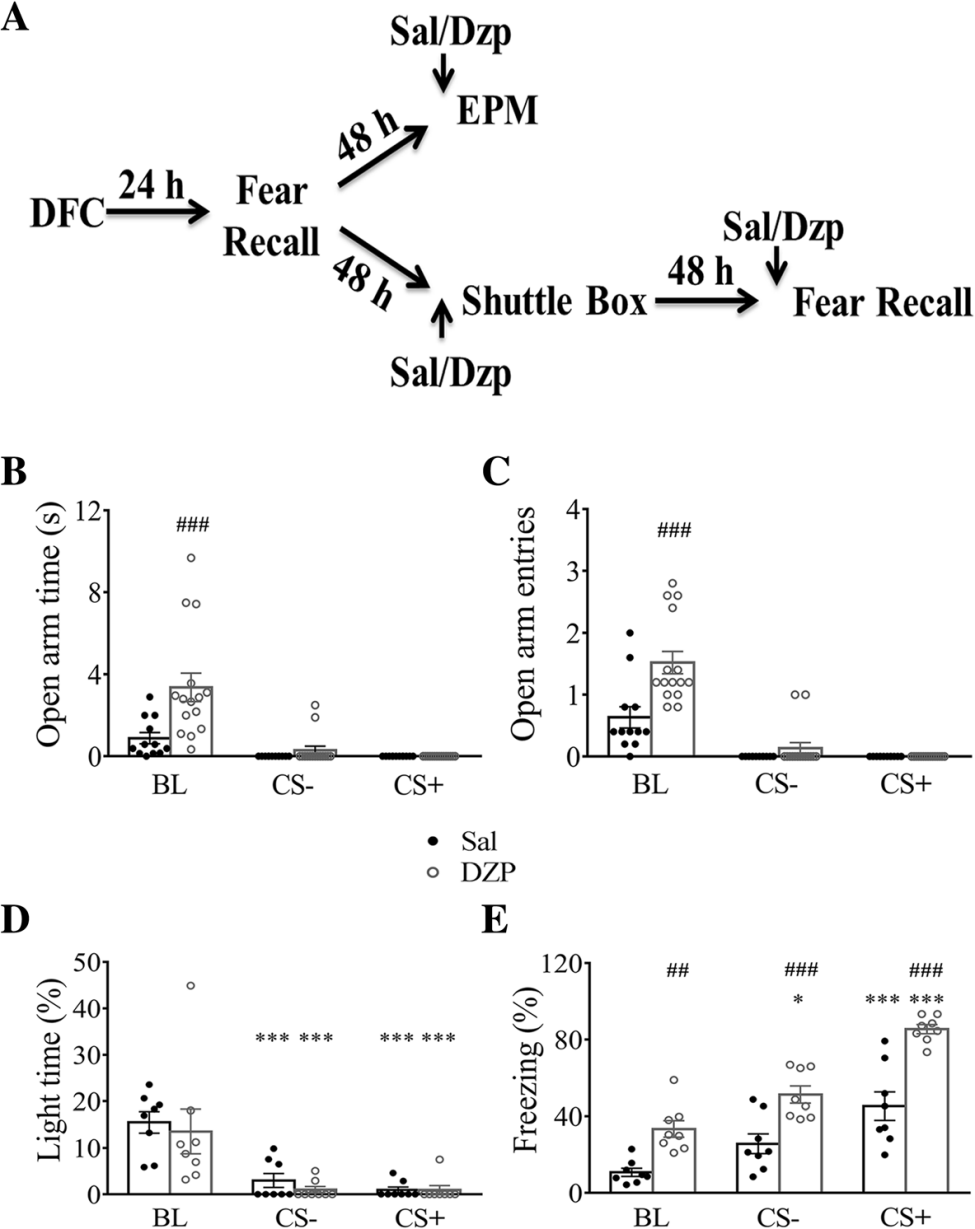
Effect of diazepam (DZP) on anxiety measurements during CS presentation. **a** Experimental procedures. Mice were divided to two groups with comparable freezing levels after fear retention test. They were tested on the EPM (saline, *n* = 12; DZP, *n* = 15) or in the shuttle box (saline, *n* = 8; DZP, n = 8) 48 h later, and received either DZP or Sal injection 30 min before the test (i.p; 1.5 mg/kg). Two days later, the shuttle box group was tested for fear retention (DZP, *n* = 8; saline, *n* = 8) with DZP or Sal injected 30 min before testing. **b** Open arm time on the EPM during CS presentation in both diazepam and saline group. **c** Number of open arm entries on the EPM during CS presentation in both diazepam and saline group. **d** Light time (%) in the shuttle box during CS presentation in both diazepam and saline group. **e** Freezing levels during fear recall in context B in both diazepam and saline group. *, *p* < 0.05; ***, *p* < 0.001; ##, *p* < 0.01; ###, *p* < 0.001. *, comparing CS to BL; #, comparing Sal to DZP. Data were analyzed by Two-way RM ANOVA with Bonferroni post-test

Next, we tested diazepam injection on anxiety measurements during CS (Fig. 2a). For EPM test (Fig. 2b), both diazepam (F _(1, 25)_ = 11.80, *p* < 0.01; two-way RM ANOVA followed by Bonferroni post-test) and CS (F _(2, 50)_ = 22.35, *p* < 0.0001) had a significant effect on open arm time. A significant effect on open arm time of drug x CS interaction was detected (F _(2, 50)_ = 7.31, *p* < 0.01). Diazepam increased open arm time significantly during BL (*p* < 0.001; Bonferroni post-test), but not during CS (*p* > 0.05) compared to saline injection. Since the majority of open arm time values were 0 for both diazepam and saline group during CS presentation, we did not perform any further analysis about CS effect on open arm time. For EPM test on open arm entries (Fig. 2c), two-way RM ANOVA followed by Bonferroni post-test analysis revealed a significant effect of diazepam (F _(1, 25)_ = 16.63, *p* < 0.001), CS (F _(2, 50)_ = 53.74, *p* < 0.0001) and drug x CS interaction (F _(2, 50)_ = 8.44, *p* < 0.001). We found that diazepam significantly increased open arm entries during BL (*p* < 0.001, Bonferroni post-test), but not during CS (*p* > 0.05), compared to saline-injection group. Since most values of open arm entries during CS were 0, we did not perform any further analysis about the effect of CS on open arm entries. For other parameters on EPM test, two-way RM ANOVA followed by Bonferroni post-test revealed that CS also induced significant changes in both diazepam group (Additional file 1; Table S3) and saline group (Additional file 1; Table S4), while diazepam had no effect during CS compared to saline group (Additional file 1: Table S5). Thus, we failed to find any changes in anxiety measurement after diazepam injection during CS on EPM. When mice were injected with diazepam prior to the shuttle box testing (Fig. 2d), two-way RM ANOVA followed by Bonferroni post-test analysis revealed that no effect of diazepam (F _(1, 14)_ = 0.31, *p* > 0.05) or drug x CS interaction (F _(2, 28)_ = 0.18, *p* > 0.05) but significant effect of CS (F _(2, 28)_ = 30.35, *p* < 0.0001). We found that CS induced a significant reductions in light time compared to BL in both diazepam (Fig. 2d, CS- and CS+ vs. BL *p* < 0.001; Bonferroni post-test), and saline group (Fig. 2d; CS- and CS+ vs. BL *p* < 0.001).

Many studies showed that diazepam reduced contextual fear response but one study showed diazepam elevated contextual and auditory fear [18–21]. There is a possibility that measurements during CS could be influenced by the occurrence of fear in the presence of diazepam. Both diazepam (F _(1, 14)_ = 58.03, *p* < 0.0001; two-way RM ANOVA followed by Bonferroni post-test) and CS (F _(2, 28)_ = 45.45, *p* < 0.0001) had a significant effect on freezing level (Fig. 2e). We found that the freezing level in diazepam-injection group during all phases of testing was significantly higher than saline group (BL, *p* < 0.01, CS-, *p* < 0.001, CS+, *p* < 0.001; Bonferroni post-test). CS+ induced a significant increase in freezing level in both diazepam group (Fig. 2e, CS+ vs. BL, *p* < 0.001) and saline group (Fig. 2e, CS+ vs. BL, *p* < 0.001), while CS- induced a significant increase in freezing level in the diazepam group (Fig. 2e, CS- vs. BL, *p* < 0.05), but not in the saline group (Fig. 2e, CS- vs. BL, *p* > 0.05). However, no significant drug x CS interaction was detected (F _(2, 28)_ = 2.11, *p* > 0.05). Hence, the influence of diazepam on freezing level could affect measurements of anxiety during CS.

Taken together, the observed increased in anxiety during CS using EPM test might reflect the occurrence of fear rather than an actual increase in anxiety. There were some subtle differences in the conditions between EPM and fear recall, such as that they took place in different rooms and CS was played back through a recorder (recorded sound) in the EPM test than directly played through a speaker during fear recall (see Methods).

### Effect of inhibiting PKMzeta activity on freezing and anxiety measurements

In the above experiments, we found evidence that occurrence of fear/freezing could impact the measurement of anxiety. However, these are indirect evidence. What we did not have in those experiments was a condition where fear was abolished or significantly reduced to allow us to examine corresponding changes in anxiety. Previous studies have found that inhibiting PKMzeta activity with a short peptide zeta interfering peptide (ZIP) led to the disappearance of fear memory [22–24]. Thus, we examine anxiety in mice injected with ZIP after fear conditioning.

**Fig. 3:**
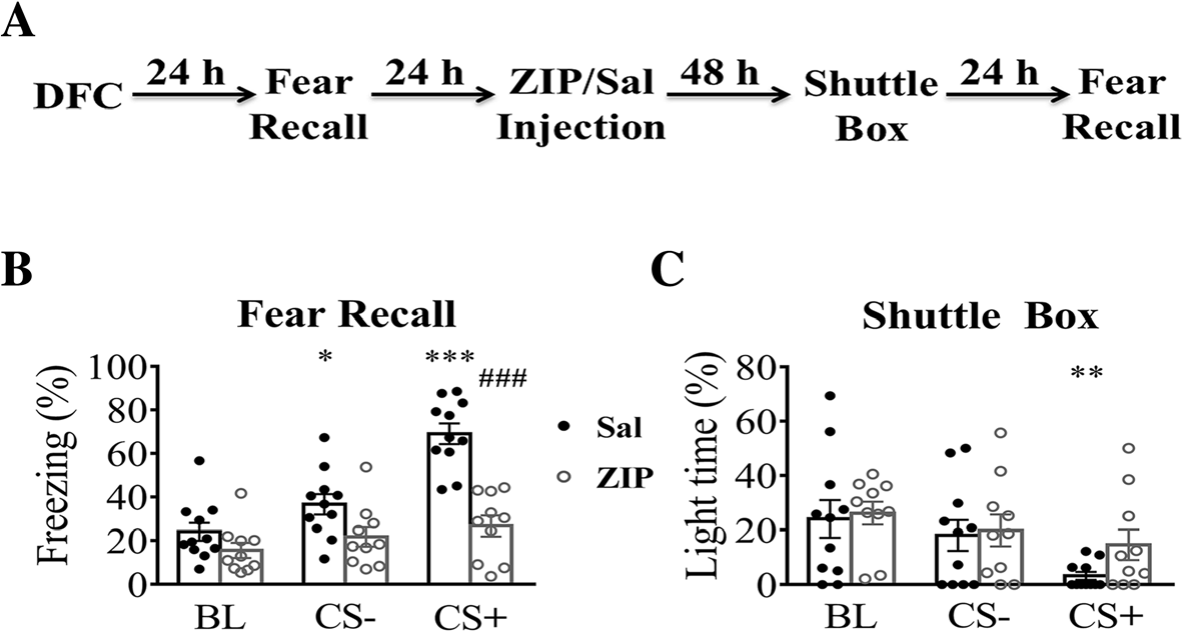
Effect of ZIP on measurements of anxiety and freezing level. **a** Experimental procedures. Mice received local injection of either saline or ZIP in the BLA. They were tested in the shuttle box in 48 h, followed by fear recall test in 24 h in context B. **b** Freezing levels during fear recall in context B in both ZIP and saline group. **c** Light time during shuttle box test in ZIP and saline group. *, *p* < 0.05; **, *p* < 0.01; ***, *p* < 0.001; ###, *p* < 0.001. *, comparing CS to BL; #, comparing saline to ZIP. Data were analyzed by Two-way RM ANOVA with Bonferroni post-test

Twenty-four hours after fear conditioning, mice were tested once for freezing levels in a non-conditioning context to establish a baseline. They were then injected with ZIP in the basolateral amygdala (BLA) in 24 h and tested again in 48 h (Fig. 3a). For freezing level test (Fig. 3b), two-way RM ANOVA followed by Bonferroni post-test revealed a significant effect of ZIP (F _(1, 19)_ = 18.72, *p* < 0.001), CS (F _(2, 38)_ = 40.53, *p* < 0.0001) and drug x CS interaction (F _(2, 38)_ =16.07, *p* < 0.0001). Freezing level in ZIP-injected mice during CS+ decreased significantly compared to the saline group (*p* < 0.001; Bonferroni post-test). CS did not induce significant freezing in ZIP-injected mice during either CS+ or CS- compared to BL (Fig. 3b, CS- and CS+ vs. BL, *p* > 0.05). In contrast, in mice injected with saline, significantly higher freezing levels were seen during both CS- and CS+ (Fig. 3b; CS- vs. BL, *p* < 0.05; CS+ vs. BL, *p* < 0.001). In shuttle box test, ZIP (Fig. 3c; F _(1, 19)_ = 1.01, *p* > 0.05; two-way RM ANOVA followed by Bonferroni post-test) had no effect on light time, but CS (F _(2, 38)_ = 6.10, *p* < 0.01) had a significant effect on light time. Both CS+ and CS- in the ZIP group had no effect on light time compared to baseline (CS-, CS+ vs. BL, *p* > 0.05; Bonferroni post-test). In the saline group, CS+ but not CS- induced a significant reduction on light time compared to baseline (CS- vs. BL, *p* > 0.05; CS+ vs. BL, *p* < 0.01). There was no significant drug x CS interaction (F _(2, 38)_ = 0.67, *p* > 0.05). Thus, when CS- and CS+ did not evoke significant freezing, the anxiety levels during the same period were also comparable to each other. These results suggest that the observed increased anxiety level during CS especially during CS+ is likely caused by occurrence of fear rather than an actual increase in anxiety. At this time, we cannot exclude the possibility that some level of anxiety remains in the ZIP-injected mice but it is below the detection of our method.

### Heart rate measurements showed a small reduction of anxiety during fear recall

**Fig. 4:**
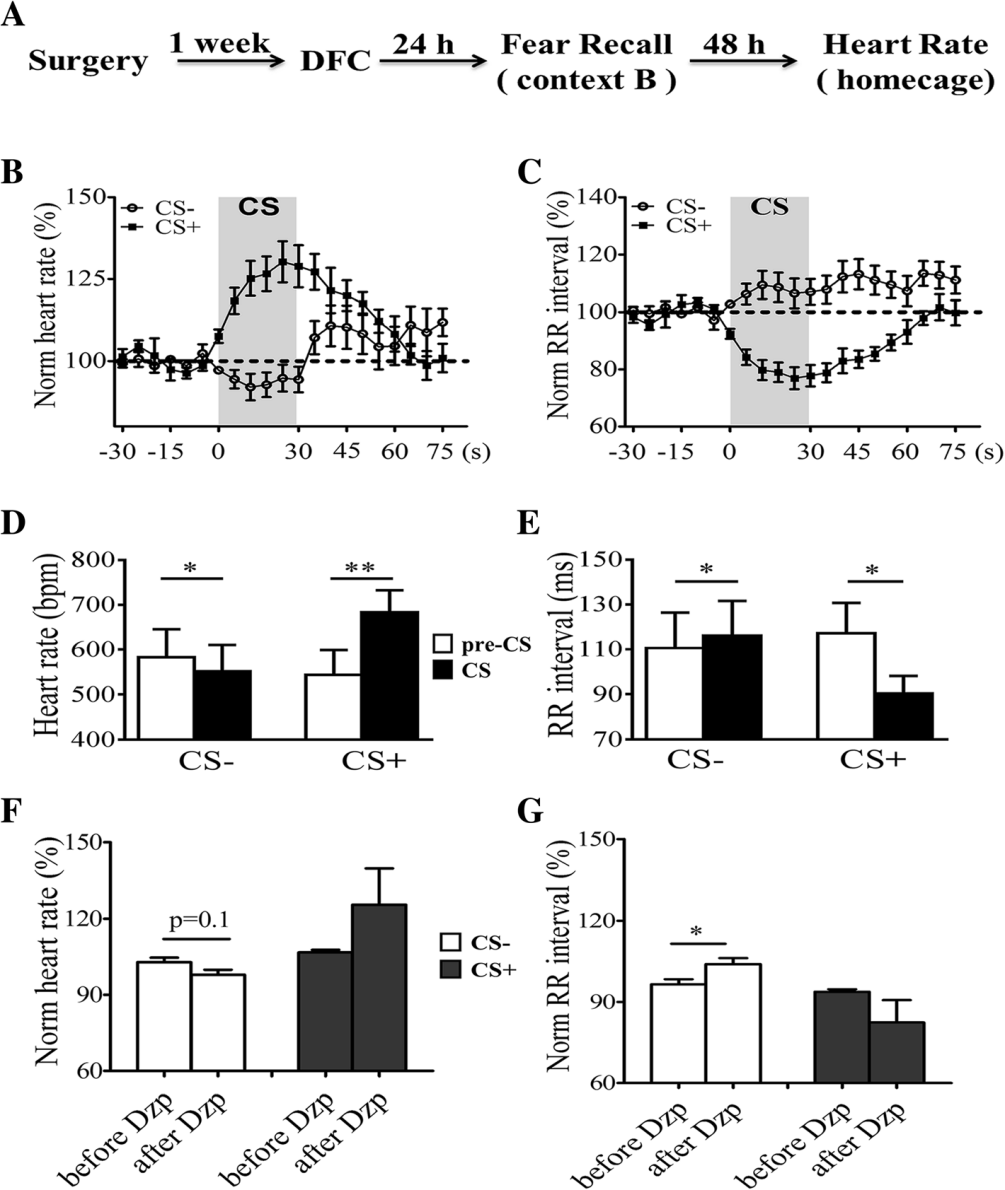
Measurement of heart rate during CS presentation. **a** Experimental procedures. **b** Normalized changes in heart rate showing a small reduction during CS- and a large increase during CS+. **c** Normalized changes in RR intervals showing a small elevation during CS- and a large reduction during CS+. Note the slow recovery towards baseline level after CS termination. **d** Quantification of changes in heart date over baseline level (pre-CS, 30 s) from data in (**b**) during the last 10 s of CS presentations. **e** Quantification of changes in RR intervals over baseline values (pre-CS, 30 s) from data in (**c**) during the last 10 s of CS presentations. **f** The effect of diazepam on heart rate during CS presentation. **g** The effect of diazepam on RR interval during CS presentation.*, *p* < 0.05; **, *p* < 0.01. Data were analyzed by Two-tailed paired t-test

In addition to behavioral measurements, changes in anxiety level readily lead to alterations in heart activity, such as altered heart rate [25–27]. Thus, we implanted mini radio-telemetry transmitter in mice to measure heart rate during fear recall (Fig. 4a). Values during a 30 s period before tone presentation was used as baseline level (BL), and HR and RR interval during CS presentations were normalized by BL. Presentation of CS- in home cage caused a small reduction in HR while CS+ caused a large elevation (Fig. 4b; *n* = 5 mice). We also calculated RR interval and found a small increase during CS- but a large reduction during CS+ (Fig. 4c). In addition, we compared changes in HR during pre-CS and CS periods. Values during a 30 s period before tone presentation was used as baseline level while values during the last 10 s of CS presentations was used for CS. A significant difference between pre-CS and CS was seen for both heart rate (Fig. 4d, pre-CS- vs. CS-, T_4_ = 3.54, *p* < 0.05; pre-CS+ vs. CS+, T_4_ = 5.94, *p* < 0.01; Two-tailed paired t test) and RR intervals (Fig. 4e, pre-CS- vs. CS-, T_4_ = 3.66, *p* < 0.05; pre-CS+ vs. CS+, T_4_ = 4.15, *p* < 0.05). Taken together, CS- induced a reduction in HR and increase in RR interval which may reflect a reduced anxiety level during CS-. To further confirm that changes in HR and RR interval indicate the anxiety state of mice, we examined whether those parameters were sensitive to diazepam. Administration of diazepam significantly decreased basal HR (Additional file 3: Figure S2A; T_3_ = 3.27, *p* < 0.05) and increased RR intervals (Additional file 3; Figure S2B; T_3_ = 3.59, *p* < 0.05). Values during a 30 s period before tone presentation was used as BL and values during the last 10 s of CS presentations were normalized by BL. Comparisons were made between normalized values. During CS- presentation, diazepam caused a small reduction in heart rates (Fig. 4f, T_3_ = 2.35, *p* = 0.10) and increased RR intervals significantly (Fig. 4g, T_3_ = 3.74, *p* < 0.05). During CS+ presentation, HR increased (Fig. 4f, T_3_ = 1.37, *p* > 0.05) while RR intervals decreased (Fig. 4g, T_3_ = 1.43, *p* > 0.05) after administrating diazepam. Freezing level during CS+ increased significantly after administrating diazepam (Additional file 3: Figure S2C; T_3_ = 4.0, *p* < 0.05) which may account for the observed changes in HR and RR interval during CS+ presentation.

### Ventral CA1 neuronal activity showed modulation by CS

All the above measurements are based on indirect measurements of anxiety and all could be confounded by potential interference from fear expression during CS presentation. Put another way, the interpretation of these results are complex and indirect. Thus, we sought to measure changes in anxiety level directly. Recent findings suggest that activity of vCA1 neurons projecting to lateral hypothalamic area (LHA) is highly related to anxiety level in mice [17] .

**Fig. 5:**
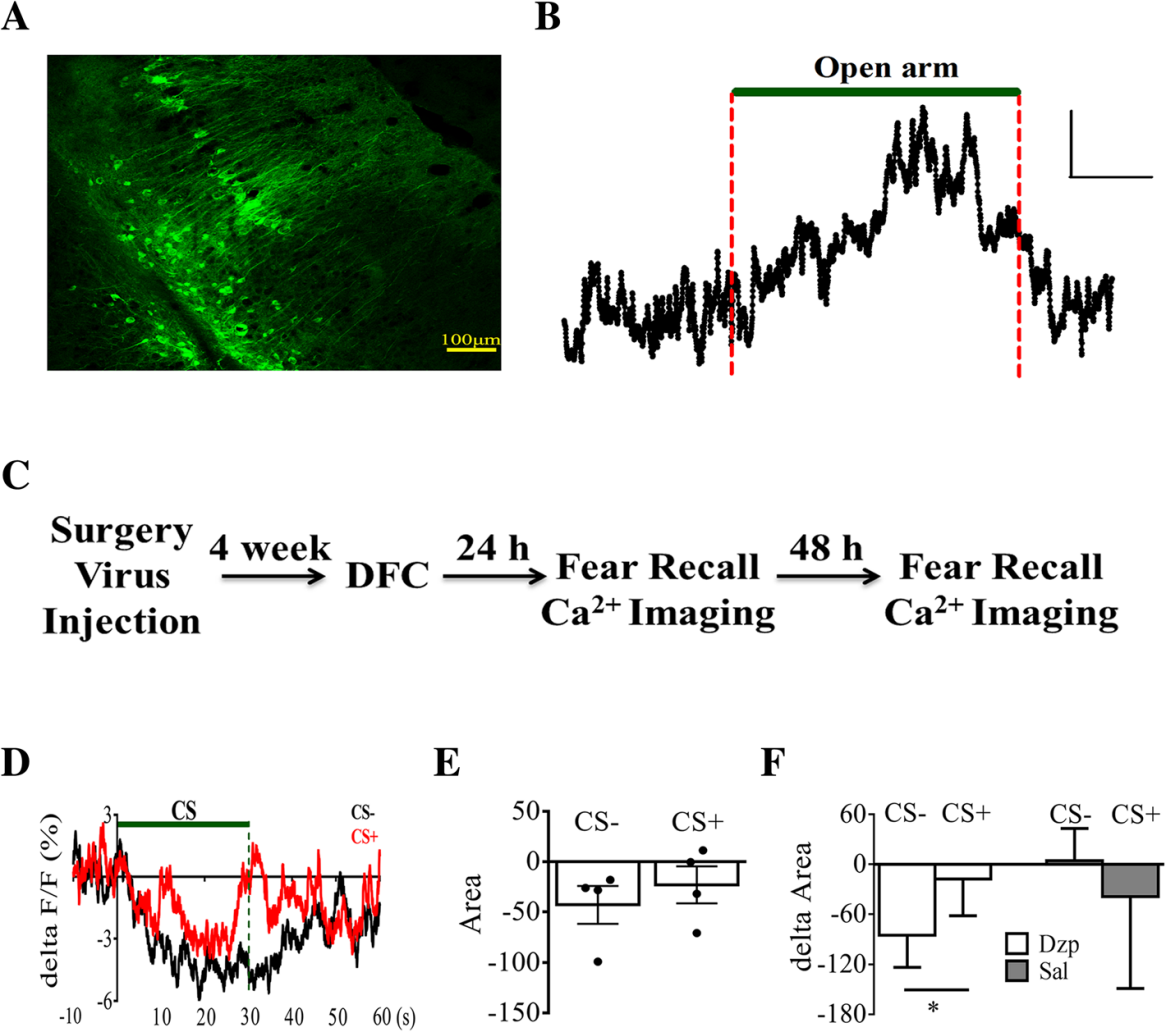
Calcium responses in vCA1 neurons that projecting to lateral hypothalamus during CS presentation. **a** Sample image of transfected neurons in vCA1. Scale bars, 100 μm. **b** Sample Ca^2+^ response showing elevation in vCA1 neurons during exploration of open arms on the EPM. Scale bars, 10% ΔF/F; 10 s. **c** Experimental procedures. **d** Averaged population Ca^2+^ responses showed gradual reduction during CS presentations which gradually recovered towards baseline after CS termination. **e** Quantification of changes in Ca^2+^ responses from data in (**d**) with integrated area from the last 10 s during CS presentations. **f** Delta integrated areas from the last 10 s during CS presentations after injection of either Dzp or Sal. Delta integrated area = integrated area after injection - integrated area before injection. *, *p* < 0.05

We expressed retrograde virus hSyn-Cre in LHA and Ef1α-DIO-GCaMP6s virus in vCA1 to examine the activity of the LHA-projecting vCA1 neurons (Fig. 5a). We found a good relationship between Ca^2+^ responses in these neurons and mouse behavior on the EPM in that significant increase in Ca^2+^ responses were detected when mice entered the open arm on EPM (Fig. 5b), confirming that the responses of neurons are associated with changes in anxiety level. We next measured responses of these neurons after discriminative fear conditioning (Fig. 5c). Ventral CA1 Ca^2+^ responses decreased slowly and steadily with the onset of CS, and were eventually lower than the baseline level at the termination of CS, and they slowly returned towards baseline (Fig. 5d). Quantification of these changes by calculating the areas under the curve during the last 10 s before CS termination showed reduction in these responses. There was no difference between CS+ and CS- (Fig. 5e; T_3_ = 0.78, CS+ vs. CS-, *p* > 0.05; Two- tailed paired t test). The reduction of Ca^2+^ activity implied that anxiety level during CS presentation is reduced. We next measured whether this reduction was sensitive to diazepam. Due to the long illumination used to obtain fluorescence signals, some level of bleaching occurred and caused a gradual and continuous reduction in the intensity of basal Ca^2+^ signal. Thus, we could not compare basal Ca^2+^ activity levels between groups to determine whether basal level anxiety has changed. During CS presentation, injection of diazepam decreased Ca^2+^ activity, but was not significant (Fig. 5f; F _(1, 5)_ = 0.23, *p* > 0.05). However, compared between CS- and CS+, diazepam caused larger reduction in Ca^2+^ signal (T_3_ = 3.72, *p* < 0.05; Two- tailed paired t test). CS+ and CS- showed no significant difference in Saline-injected group (T_2_ = 0.42, *p* > 0.05; Two- tailed paired t test).

## Discussion

In a series of experiments, using various methods established for measuring anxiety in rodents, we examined whether anxiety levels are modulated during CS presentation in mice received differential fear conditioning. We found: (1) anxiety measurements using shuttle box and EPM are heavily influenced or confounded by the occurrence of fear and hence cannot be used with confidence to draw conclusions. (2) In mice injected with diazepam, basal anxiety level was reduced demonstrating anxiolytic efficacy but such effect was absent during CS. (3) Inhibition of PKMzeta activity via injection of ZIP into BLA abolished fear responses and reduced anxiety level to baseline (pre-CS) level during CS. (4) Measurement of heart rates suggests a small reduction in anxiety level during CS- (in the absence of fear responses). (5) Ca^2+^ responses in vCA1 neurons which track changes in anxiety level revealed a reduction during CS which may suggest reduced anxiety level and this reduction can be enhanced by diazepam. Thus, we conclude that there is likely a *transient* reduction in anxiety level during CS presentation in fear-conditioned mice.

In general, anxiety is postulated to occur to potential threat in a pre-encounter defense manner to prepare an animal to respond based on the nature of the incoming stimulus and prior experience/memory of similar encounters. In contrast, fear is usually viewed as a response to acute and present threat for a post-encounter defense in order to deal with the current situation [1]. In addition, fear is also viewed as triggered by stimuli more concrete and specific while anxiety by stimuli more diffuse and abstract. Although some symptoms of fear and anxiety are difficult to distinguish from each other, some studies have shown that distinct neural circuitries for fear and anxiety. In general dorsal DG control contextual fear learning and ventral DG show correlation with anxiety [28]. The vCA1-BA projecting neurons modulate fear memory encoding and retrieval whereas vCA1-LHA projecting neurons control anxiety-related behavior [17]. Distinct hippocampal microcircuits underlie or modulate the expression of fear versus anxiety [29]. In this study, we asked a simple question: can we observe any change in the anxiety level during presentation of a conditioned stimulus (CS) which is associated with foot shock after fear conditioning.

From behavioral analysis it is difficult to determine whether anxiety level is altered significantly during CS presentation. This is likely caused by: (1) the substantial impact of fear expression on measuring anxiety under our experimental conditions precludes an accurate measurement of anxiety. With high fear expression (as during CS+), responses predominantly reflect the occurrence of freezing, and anxiety measurements are not valid. In other words, what is measured is mostly fear rather than anxiety. (2) The likely different sensitivity of measurements in fear and anxiety. Even under situations where fear is quite low, potential differences in freezing and anxiety might reflect the differential sensitivity in their respective measurements rather than actual differences. For example, it has been suggested that measuring fear using freezing is not as sensitive as measuring fear-induced suppression of food intake [30]. Furthermore, it is possible that this difference in sensitivity might be different for each animal.

Diazepam exhibited its expected anxiolytic efficacy during baseline in the fear-conditioned mice on EPM, suggesting an elevated innate anxiety level which may be similar to generalized anxiety in human. However, diazepam did not reduce anxiety during CS when behavior was measured. It is possible that during CS, fear responses overwhelmed the behavior of mice on EPM so that any anxiolytic effect of diazepam could not be revealed since it was below the detection threshold. Alternatively, this lack of efficacy might be due to reduced GABAa receptor density and reduced GAD 67 and extracellular GABA level in the BLA after fear conditioning [31–33]. On the other hand, we did find some evidence for reduced anxiety by diazepam during CS- as measured using heart rate. Interestingly, this reduction was not seen during CS+ which we suggested to be caused by large increase in fear responses which also altered heart rate measurements.

Mice that received ZIP injection in BLA had their fear responses reduced to baseline level, consistent with prior reports [22–24]. PKMzeta activity appears to regulate anxiety level in mice, but it is unclear whether a selective reduction of PKMzeta activity in the BLA is sufficient to reduce anxiety [34, 35]. It has been shown that excitation/inhibition balance in BLA affects anxiety level and hence it will be interesting to further test the possibility that PKMzeta might affect anxiety by altering E/I balance in BLA in the follow up experiments.

Emotional states such as anxiety have been shown to be related to cardiovascular function [36]. Therefore, we examined heart rate and RR intervals during CS presentation. We found a small reduction in heart rate and increase in RR intervals during CS- when mice were in their home cages. Since there was no fear (freezing) during CS-, we suggest that this change reflects reduced anxiety level during CS-. The reason why we did not find any reduction during CS+ is the strong interference of fear responses, based on prior studies showing a significant HR increase with auditory stimulus paired with a foot shock [37, 38].

The main reason for the uncertainty and difficulty in reliably detecting changes in anxiety level using the above methods is that many factors contribute to and influence the final readout, and in essence we are not reading out anxiety directly. To circumvent this limitation, we examined vCA1 Ca^2+^ responses which have been shown to reflect changes in anxiety levels [17]. These experiments showed a rather surprising, unexpected finding in that we found a reduction of anxiety signals during both CS+ and CS-. We propose that CS- acts a safety cue to reduce anxiety, which is supported by prior findings that safety cues have anti-depressant function and can serve to improve the flexibility of animals’ behavior [39] . We also suggest that CS+ signals the sure occurrence of expected foot shock to reduce anxiety. There is a 100% association (absolutely certainty) between CS+ and unconditioned stimulus (US, foot shock) as determined by the conditioning protocol, and hence mice learn to expect the imminent arrival of US with the onset of CS+. It has been shown that fear reactivation caused elevation in the plasma corticosterone level and is associated with reduced anxiety level (increased open arm entries), and injection of corticosterone mimicked this reduction in anxiety [40]. The general conclusion is similar to ours but of a different time scale.

The hippocampus has a critical role in both mnemonic and emotional functions. Studies have suggested that hippocampus to have separate functional domains [41, 42], in that dorsal hippocampus (dHPC) is necessary for spatial memory while vHPC necessary for emotional functions [14, 43–46]. Some studies have shown that vHPC is involved in auditory fear conditioning since pre-training or post-training lesions of vHPC disrupted the acquisition and expression of auditory fear conditioning [47–49]. Most studies have shown a positive correlation between anxiety level and vHPC activity, optogenetic or pharmacological manipulation of vHPC directly affects anxiety-related behavior [16, 17, 50, 51]. Dense projections from vHPC to other brain regions are likely involved in anxiety, such as hypothalamus, and amygdala and medial prefrontal cortex [16, 17, 50, 52–54]. A recent study has showed that vCA1-LHA pathway is a direct route to influence anxiety behavior [17]. Our finding of reduced Ca^2+^ responses suggests a reduction of CS-induced anxiety level.

In conclusion, we have provided evidences that are consistent with conditioned stimulus-triggered reduction in anxiety level in animals that have undergo aversive experiences. We do acknowledge that these are preliminary evidences and subject to alternative interpretation. We suggest multiple approaches, including behavioral, pharmacological, and in vivo recording of neuronal activity, need to be used for a clear distinction between anxiety and fear. Among them, further dissection of the underlying circuitry is of the highest priority.

## Additional files

Additional file 1: Other parameters on EPM test. **Table S1.** Other parameters in EPM during CS presentation. **Table S2.** Other parameters in EPM about the effect of diazepam on innate anxiety of fear-conditioned mice. **Table S3.** Other parameters in EPM in diazepam group on anxiety measurements during CS presentation. **Table S4.** Other parameters in EPM in saline group on anxiety measurements during CS presentation. **Table S5.** Other parameters in EPM compare saline with diazepam on anxiety measurements during CS presentation. *, *p* < 0.05; **, *p* < 0.01; ***, *p* < 0.001. Data were analyzed by One-way or Two-way RM ANOVA with Bonferroni post-test. (XLS 31 kb)

Additional file 2 **Figure S1.** Effect of diazepam on innate anxiety in fear-conditioned mice (A) Experimental procedures. Twenty-four hours after discriminative fear conditioning (DFC), mice were separated into two groups with comparable freezing levels. In 48 h, one group was tested in the shuttle box and OFT (saline, *n* = 13; DZP, n = 13), while the other group on the EPM (saline, *n* = 12; DZP, *n* = 15). DZP or saline (1.5 mg/kg) was injected i.p 30 min before testing in the shuttle box, OFT or EPM. (B) DZP significantly increased open arm time on EPM during BL (Two-tailed unpaired t test, T_25_ = 3.03, *p* < 0.01). (C) DZP significantly increased the number of entries to open arm on EPM (Two-tailed unpaired t test, T_25_ = 3.49, *p* < 0.01). (D) DZP had no effect on the light time (%) measured in shuttle box (Two-tailed unpaired t test, T_24_ = 0.20, *p* > 0.05). (E) DZP had no effect on the center time in the OFT (Two-tailed unpaired t test, T_24_ = 1.45, *p* > 0.05). (F) DZP had no effect on the total distance in OFT (Two-tailed unpaired t test, T_24_ = 1.47, *p* > 0.05). **, *p* < 0.01. Data were analyzed by two-tailed unpaired t-test. (TIF 2224 kb)

Additional file 3 **Figure S2.** Effect of diazepam on heart rate in fear-conditioned mice. (A) DZP significantly decreased heart rate during BL (Two-tailed paired t test, T_3_ = 3.27, *p* < 0.05). (B) DZP significantly increased RR interval during BL (Two-tailed paired t test, T_3_ = 3.59, *p* < 0.05). (C) DZP significantly increased freezing level during CS+ presentation (Two-tailed paired t test, T_3_ = 4.0, *p* < 0.05).*, *p* < 0.05. Data were analyzed by Two-tailed paired t-test. (TIF 1514 kb)

## Abbreviations

BLA: basolateral amygdala
bpm: beats per minute
CS: conditioned stimulus
DFC: discriminative fear conditioning
dHPC: dorsal hippocampus
DZP: diazepam
EPM: elevated plus maze
HR: Heart rate
ITI: intertrial interval
LHA: lateral hypothalamus area
OFT: open field test
PKMzeta: protein kinase Mzeta
R-R intervals: beat-to-beat interval
Sal: saline
US: unconditioned stimulus
vCA1: ventral hippocampal CA1
vHPC: ventral hippocampus
ZIP: zeta inhibitory peptide
