# Supplementary figures and images for: Conditioned stimulus presentations alter anxiety level in fear-conditioned mice
Source: Mol Brain. 2019 Mar 29;12:28. doi: 10.1186/s13041-019-0445-4 (PMC6441152; doi:10.1186/s13041-019-0445-4)

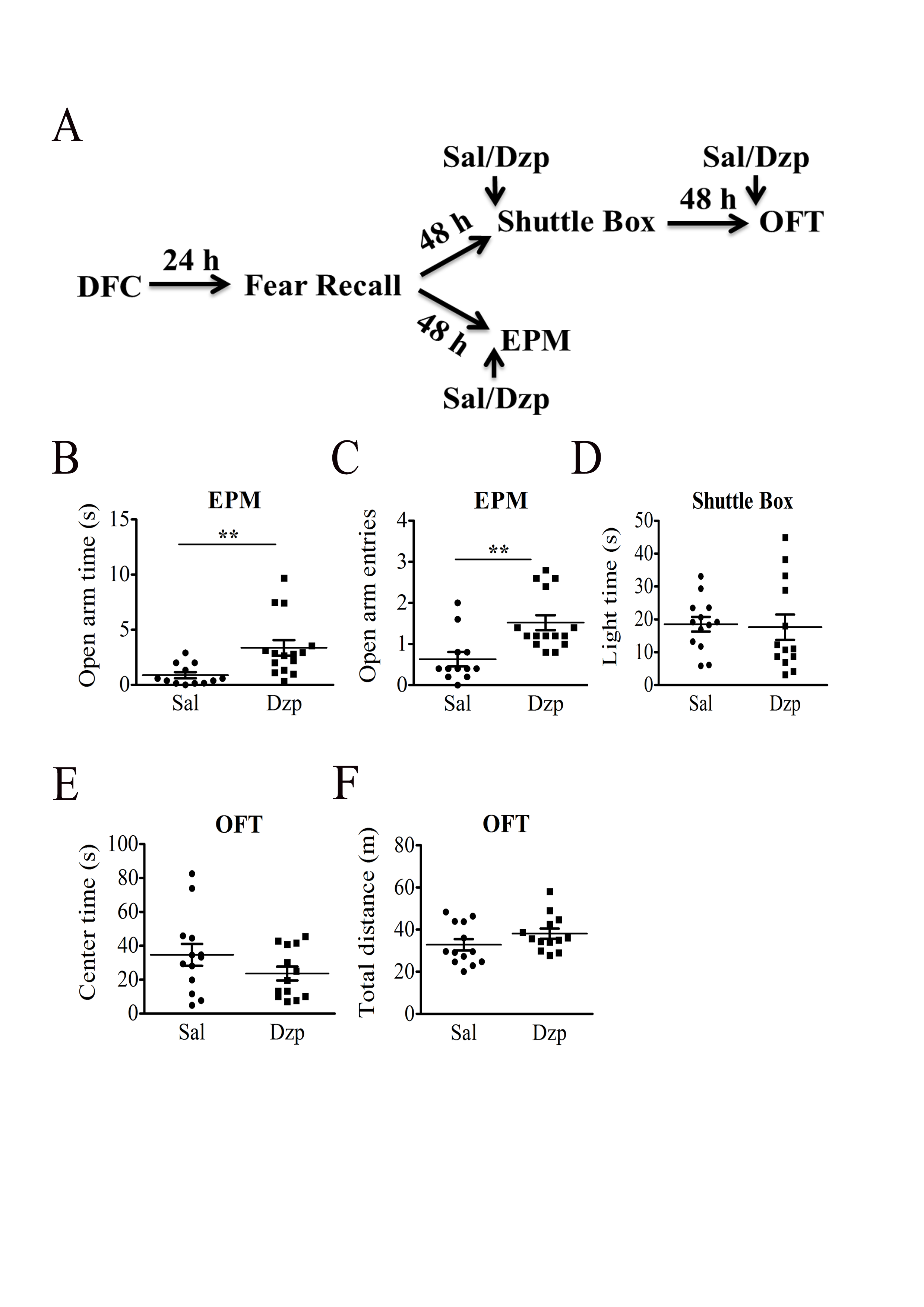

Supplement: Supplementary file 2 — Figure S1. Effect of diazepam on innate anxiety in fear-conditioned mice (A) Experimental procedures. Twenty-four hours after discriminative fear conditioning (DFC), mice were separated into two groups with comparable freezing levels. In 48 h, one group was tested in the shuttle box and OFT (saline, n = 13; DZP, n = 13), while the other group on the EPM (saline, n = 12; DZP, n = 15). DZP or saline (1.5 mg/kg) was injected i.p 30 min before testing in the shuttle box, OFT or EPM. (B) DZP significantly increased open arm time on EPM during BL (Two-tailed unpaired t test, T25 = 3.03, p < 0.01). (C) DZP significantly increased the number of entries to open arm on EPM (Two-tailed unpaired t test, T25 = 3.49, p < 0.01). (D) DZP had no effect on the light time (%) measured in shuttle box (Two-tailed unpaired t test, T24 = 0.20, p > 0.05). (E) DZP had no effect on the center time in the OFT (Two-tailed unpaired t test, T24 = 1.45, p > 0.05). (F) DZP had no effect on the total distance in OFT (Two-tailed unpaired t test, T24 = 1.47, p > 0.05). **, p < 0.01. Data were analyzed by two-tailed unpaired t-test. (TIF 2224 kb) [file 13041_2019_445_MOESM2_ESM.tif]

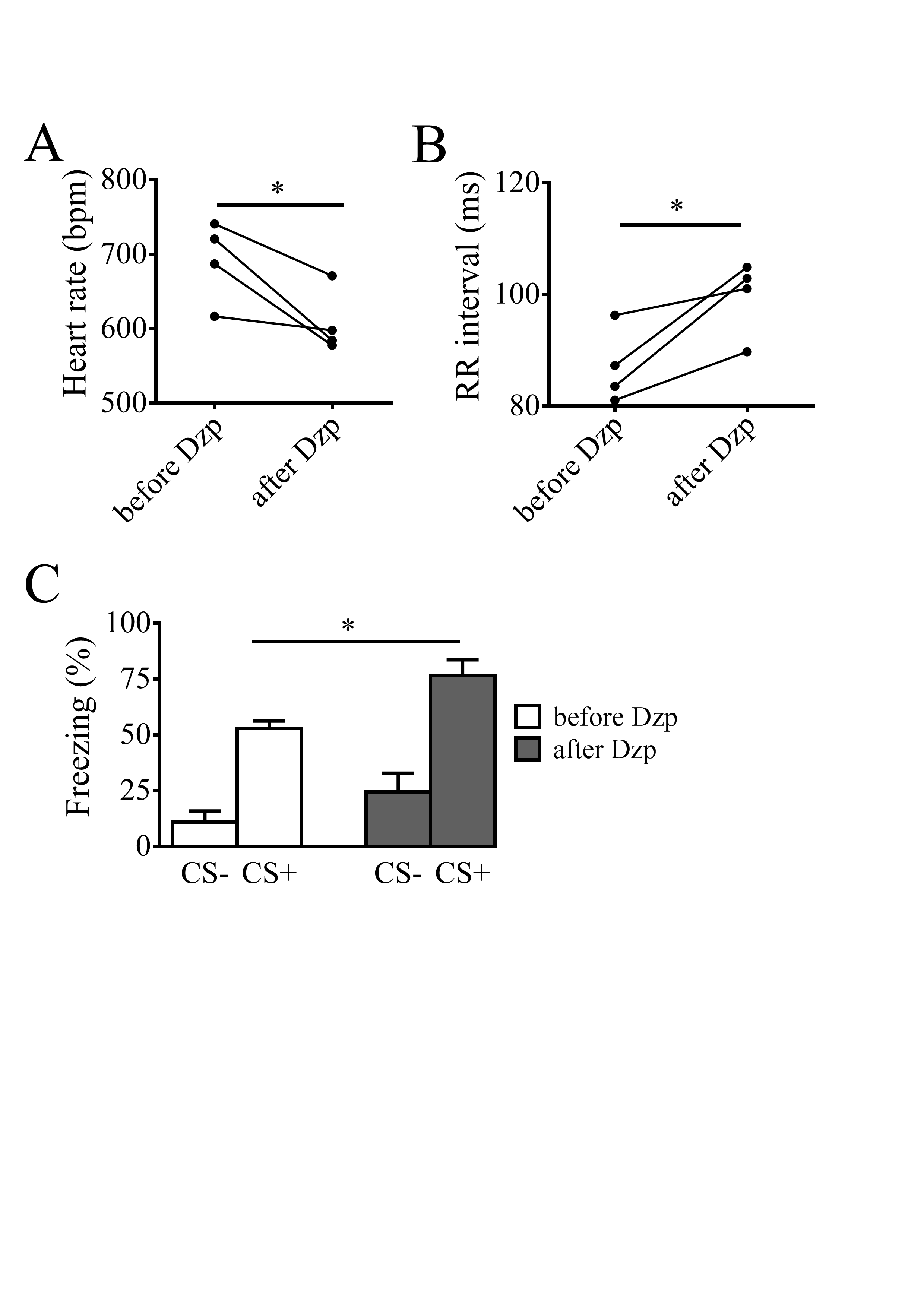

Supplement: Supplementary file 3 — Figure S2. Effect of diazepam on heart rate in fear-conditioned mice. (A) DZP significantly decreased heart rate during BL (Two-tailed paired t test, T3 = 3.27, p < 0.05). (B) DZP significantly increased RR interval during BL (Two-tailed paired t test, T3 = 3.59, p < 0.05). (C) DZP significantly increased freezing level during CS+ presentation (Two-tailed paired t test, T3 = 4.0, p < 0.05).*, p < 0.05. Data were analyzed by Two-tailed paired t-test. (TIF 1514 kb) [file 13041_2019_445_MOESM3_ESM.tif]
